# Forgetting “Novel” but Not “Dragon”: The Role of Age of Acquisition on Intentional and Incidental Forgetting

**DOI:** 10.1371/journal.pone.0155110

**Published:** 2016-05-10

**Authors:** Alejandra Marful, Carlos J. Gómez-Ariza, Analía Barbón, Teresa Bajo

**Affiliations:** 1 University of Jaén, Jaén, Spain; 2 International University of La Rioja, Logroño, Spain; 3 Mind, Brain and Behavior Research Center, University of Granada, Granada, Spain; University of Akron, UNITED STATES

## Abstract

Two experiments studied how the age at which words are acquired (Age of Acquisition, AoA) modulates forgetting. Experiment 1 employed the retrieval-practice paradigm to test the effect of AoA on the incidental forgetting that emerges after solving competition during retrieval (i.e., retrieval-induced forgetting, RIF). Standard RIF appeared with late-acquired words, but this effect disappeared with early-acquired words. Experiment 2 evaluated the effect of AoA on intentional forgetting by employing the list-method directed forgetting paradigm. Results showed a standard directed forgetting effect only when the to-be-forgotten words were late-acquired words. These findings point to the prominent role of AoA in forgetting processes.

## Introduction

Skills and concepts learned during early childhood seem to have a special status in cognition. Thus, the ease with which one specific representation is processed can be determined by the age at which it was acquired (Age of Acquisition, AoA). For example, certain musical abilities, such as absolute pitch, can usually only be learnt during childhood [1], and concepts that are acquired early in life are processed faster than late-acquired ones [2–3].

An advantage for early-acquired words has been reported in many psycholinguistic domains such as picture naming, word reading, and auditory and visual lexical decision tasks [3–6]. In addition, early-acquired words have been shown to be more resistant to cognitive impairment in Alzheimer’s disease and other neuropsychological problems [7–8]. This advantage for early-acquired representations has been attributed, for example, to better connections in semantic memory [9,10]. It has also been suggested that early-acquired words constitute the basis by which the mental network is organized. Accordingly, once a mental configuration is created with these first representations, it losses plasticity so that later acquired representations are more difficult to integrate ([11], see also [12–14] for a review and for alternative hypotheses).

Despite its relevance for cognition, most of the studies on AoA have primarily focused on the language domain. The link between AoA and other cognitive functions such as episodic memory has been much less investigated, with available studies providing mixed results [15–18]. One intriguing question in memory research is why we can easily forget some information while other memories seem to be indelible. Given the special status of early-acquired knowledge representations, we aimed to explore whether AoA plays a role in forgetting. Thus, we tested the effect of AoA on two types of forgetting phenomena that are thought to be aftereffects of exerting control over unwanted or irrelevant memories [19]. In Experiment 1, we focused on retrieval-induced forgetting (RIF), an incidental type of forgetting whereby practicing retrieval (retrieval-practice, RP) of some studied items causes the temporary inaccessibility of related non-target items that compete for retrieval. From an influential standpoint [20, 21], competing memories during retrieval are subjected to inhibitory control which helps facilitate the retrieval of target memories. Hence, if once non-target traces turn out to be relevant later, as is the case in the RP procedure, they become less recallable because of the residual effect of inhibition. In Experiment 2, we focused on list-method directed forgetting (LM-DF), an intentional type of forgetting that is normally observed after providing participants with the instruction to forget previously encoded items. According to a well-established account (for an alternative account, see [22]), directed forgetting is caused by a transitory state of inhibition of the to-be-forgotten items which is triggered to prevent these items from hindering learning/recall of new items [23–24]. While differing in a variety of aspects, RIF and LM-DF are thought to reflect the adaptive value of making unwanted information less accessible.

On the basis of the previously described cognitive advantages of early-acquired words, we expected early-acquired items to be more resistant to both intentional and incidental forgetting than later-acquired ones. Our hypothesis is that because early acquired words seem to be better interconnected and integrated in memory, they should be less susceptible to both incidental and intentional forgetting effects.

## Experiment 1

RIF has been widely studied using the retrieval-practice procedure [21]. In this paradigm, category-exemplar pairs are presented for study (fruit-orange, fruit-apple, etc.). Then, during a retrieval-practice phase, participants are cued to repeatedly recall half of the items from half the categories (practiced items, Rp+; fruit ap____). Finally, participants’ memory of all the studied items is tested. Results typically show that unpracticed items from practiced categories (Rp- items; orange) tend to be more poorly recalled than control (Nrp) items (RIF effect). Whereas different mechanisms have been posited to account for RIF (see [25] for a context-change account), memory inhibition has emerged as a potential candidate [21, 26–28]. From this viewpoint, the memory impairment for competitor (Rp-) items is interpreted as the aftereffect of their inhibition during retrieval practice.

Starting from the premise that early-acquired words hold a special status in cognition, and, regardless of the mechanism underlying RIF, one would expect these words, relative to late-acquired ones, to be less prone to incidental forgetting. This expectation might seem at odd with the frequency effect reported by Anderson, Bjork, and Bjork (1994) [29]. In their experiments they manipulated the category frequency of the competitor exemplars and reported larger RIF effects for high than low frequency words, suggesting that highly accessible representations are more vulnerable to retrieval-induced forgetting. Note, however, that category frequency in Anderson et al’s study refers to the strength of the category-exemplar connections, with high frequency exemplars being associated with higher probabilities of being activated upon the category presentation. This would in turn produce stronger competition relative to weaker elements with proportional suppression acting upon these competing representations. In contrast, although early representation may also be more accessible, we assume that they are more resistant to forgetting because their internal structure (more interconnected or better consolidated) protect them from competition and forgetting. Supporting this hypothesis, previous studies have shown that conditions fostering integration through instructions [30], or by the nature of the materials [31–33] reduce forgetting effects.

### Methods

#### Participants

Forty Spanish students (36 women; M = 20.07, SD = 2.70) from the universities of Granada and Jaén participated in this experiment in exchange for course credit. Sample size was determined on the basis of prior studies [34]. The study was approved by the local ethics committees (Universities of Jaén and Granada). Participants gave written informed consent.

#### Materials

Eight orthographically based categories (i.e., semantically unrelated words that started with the same two letters: CA-camino (path), CA-cachorro (puppy), etc.; see [34]) each including six words were created from Davies et al. [4] database. Half of them were composed of early-acquired words (i.e., 24 words), whereas the other half comprised late-acquired words (i.e., 24 words).

Late- and early-acquired lists (see S1 Table) differed in age of acquisition scores [4] *F*(1,46) = 134.67, *p* < .0001, *η*^2^ = .74 but were matched in word frequency [35] from the NIPE database [36] *F*(1,46) = .36, *p* = .55, *η*^2^ 008, number of phonological *F*(1,46) = 2.287, *p* = .14, *η*^2^ .05 and orthographic *F*(1,46) = 3.67, *p* = .06, *η*^2^ = .07 neighbors, number of words beginning with the first two letters *F*(1,46) = 2.085, *p* = .16, *η*^2^ = .04 and number of letters *F*(1,46) = .009, *p* = .92, *η*^2^ = .00 but differed in concreteness *F*(1,46) = 15.3, *p* <. 0001, *η*^2^ = .25 [37]. Semantic and associative relationships among words were kept to a minimum; there was no phonological overlap between the first two letters of the words from the different categories, and all words were between two and five syllables. Two more six-words sets were created to be used as fillers.

For the final memory test, two or three letters (depending on the word length) of each word were removed, with one of these always being the first letter. We did this to minimize retrieval interference from Rp+ over Rp- items [21].

#### Procedure

Participants were instructed to study a list of unrelated words sharing the first two letters for a later memory test. To avoid floor effect due to very long lists, early-acquired lists were presented to half of the participants and late-acquired lists to the remaining students. Each item was presented individually next to its first two letters (e.g., CA-camino) in the center of the screen (5000 ms), preceded by a blank screen (1000 ms) and a fixation point (1200 ms) and followed by a blank screen (500 ms). The complete list of stimuli was presented twice for study in a random order. Six filler items appeared at the beginning and 6 at the end of the study phase to minimize primacy and recency effects. During the retrieval-practice phase participants performed repeated retrieval of half of the items from half of the categories (12 Rp+ items). The practiced items and, consequently, the items that served as Rp- and Nrp- varied across two counterbalance conditions. Following a pre-cueing procedure [34], a fixation point appeared (1200 ms), followed by the lexical category cue (CA) (2000 ms), a blank screen (1000 ms), and the word stem (car) for retrieval (6000 ms). Each Rp+ item appeared four times.

After a five-minute distractor task participants performed an unexpected final memory test where fragments of the previously studied words were presented for retrieval (*-am-no* to retrieve *camino*). Each fragment (8000 ms) was preceded by a fixation point (1200 ms) and followed by a blank screen (1000 ms). To minimize output interference, fragments corresponding to the unpracticed (Rp- and Nrp-) items appeared before those fragments corresponding to the practiced items.

#### Design

The experiment used a 2 (item type: Rp-, Nrp-; within-participants) X 2 (age of acquisition; early-, late-acquired words; between-participants) mixed-factorial design. Notice that since two counterbalanced conditions were created, Rp- items in one condition were compared to the same items that served as baseline (Nrp-) items in the other condition (the same type of correspondence occurred for Rp+ and Nrp+ items).

### Results

#### Retrieval practice

Average recall success did not differ between conditions (late condition, M = .56, SD = .22; early condition, M = .46, SD = .22), *F*(1,38) = 2.14, *p* = .15, *η*^2^ = .05).

#### Final test

A 2 (item type: Rp-, Nrp-) x 2 (AoA: early, late) repeated measures ANOVA showed that the main effect of item type was not significant, *F*(1,38) = 1.16, *p* = .29, $\eta_{p}^{2}$ = .03. See Table 1. More importantly, the main effect of AoA *F*(1,38) = 13.85, *p* = .001, $\eta_{p}^{2}$ = .27 was modulated by the item type x AoA interaction *F*(1,38) = 4.86, *p* = .03, $\eta_{p}^{2}$ = .12. Thus, while late-acquired words showed the standard RIF effect (competitor, Rp-, items were more poorly recalled than baseline, Nrp-, items, *F*(1,19) = 5.48, *p* = .03, *η*^2^ = .22, RIF was not present in the early-acquired words condition *F*(1,19)<1, *η*^2^ = .03.

**Table 1:** Final memory test recall (standard deviations in brackets) in Experiment 1.

	Rp+	Nrp+	Rp-	Nrp-	RIF effect (Rp-) -(Nrp-)
Late	.78 (.22)	.73 (.17)	.49 (.24)	.66 (.21)	-17
Early	.71 (.24)	.70 (.30)	.78 (.18)	.73 (.26)	5

Although not relevant for our hypothesis, for completeness we also checked for facilitation effects by means of a 2 (item type: Rp+, Nrp+) x 2 (AoA: early, late) repeated-measures ANOVA (see Table 1 and S2 Table). Neither the main effects nor the interaction (all with *F* < 1) reached statistical significance. This lack of overall facilitation is consistent with previous studies that have also employed independent cues (i.e., memory cues different than the ones used to do retrieval practice) during the final memory test, and suggest that unlike forgetting, the benefit of retrieval practice may depend on cue-overlap between the practice phase and the memory test [38–39]. Note also that to minimize their influence over Rp- items’ recall, the cues for Rp+ items appeared at the end of the memory test and this could also play a role in reducing facilitation.

### Discussion

Experiment 1 revealed that while the standard RIF effect was observed with late-acquired words, there was no forgetting effect with early-acquired ones. Hence, the early acquisition of a word seems to protect it from incidental forgetting, at least during repeated retrieval of related items. This finding suggests that early-acquired representations, which are assumed to be strongly consolidated and overlearned, play a prominent role in cognition possibly because they are better interconnected and more efficiently represented in the memory system (see, for example, [12–14] for a review of the AoA explanatory hypothesis).

As mentioned, although at first glance the absence of RIF for early-acquired words might seem at odd with previous literature showing larger RIF effects for high-frequency words [29], it is consistent with studies reporting that RIF only emerges if Rp- items compete with Rp+ items during retrieval practice and it is reduced when items integration is promoted (e.g., [34,40]). In this line, previous studies have shown that the memory impairment for Rp- items decreases, disappears or is less durable in experimental settings when the nature of the to-be-studied materials induces the building of associative or semantic links [31–33], or when participants (spontaneously or following the experimenter’s instructions) establish these connections [30]. Hence, we posit that, unlike high-frequency words, early-acquired words, which are highly interconnected and better represented in the memory system, are less susceptible to inhibition and forgetting during retrieval practice.

The absence of RIF in the early AoA condition does not seem to be related to a deflated baseline condition. In fact, early-acquired Nrp- items were better recalled than late-acquired Nrp- items, with performance in both control conditions within the range of previous RIF studies [41]. However, it could be argued that the observed differences between early and late acquired words regarding RIF could be explained in terms of resources availability. This argument would suggest that retrieval of early-acquired words could involve less cognitive resources (less cognitive load) than late- acquired ones and, as a consequence, no forgetting would show up. Contrary to this argument, however, previous findings indicate that it is precisely the presence of high-cognitive load conditions what may prevent RIF from emerging [42–43]. Because selective retrieval is thought to place demands on attentional control to overcome interference from competing memories [20], less cognitive resources available during retrieval practice would make inhibition harder to implement. In our experiment, however, it was the late AoA condition (presumably the one demanding the highest cognitive load) that exhibited the aftereffect of inhibitory control (RIF).

## Experiment 2

In order to explore whether the AoA effect generalizes to other situations requiring memory control, in Experiment 2 we examined the role of AoA in intentional forgetting by means of the LM-DF paradigm [23]. A first list of items (L1) was presented for study followed by a cue to either recall (remember group) or forget (forget group) the studied list. Participants then studied a second list (L2) and, finally, memory of List 1 was tested. The forget group typically exhibits a memory impairment for List 1 relative to the remember group. This directed-forgetting effect (DF) has been attributed to a transitory state of inhibition of the to-be-forgotten list triggered by the instruction to forget [19, 23–24]; for a non-inhibitory account, see [22]. To assess whether AoA may also modulate the DF, a list of early- or late-acquired words was presented for study followed by either recall or forget instructions. To the extent that AoA may diminish intentional forgetting, we expected larger DF effects with late-acquired than early-acquired words.

### Methods

#### Participants

Seventy-six Spanish students (68 women; M = 21.21, SD = 3.7) from the University of Jaén participated in exchange for course credit. Data from one participant in the forget condition were replaced because he anticipated the L1-memory test. Sample size was determined on the basis of prior studies [44]. The study was approved by the local ethics committee (University of Jaén). Participants gave written informed consent.

#### Materials

Three lists of ten words were created: an early-acquired word list and a late-acquired word list which served as L1 in the early- and late-acquired condition, respectively; and a third list that functioned as L2 in both conditions. Semantic relations between items in each list were kept to a minimum.

Items from L1s (see S1 Table) were selected from Experiment 1 materials. AoA values were lower in the early-acquired list than in the late-acquired list [*t*(18) = -7.9, *p* < .0001, *d* = 3.64]. L1s did not vary in frequency *F*(1,18) = .00, *p* = .99, *η*^2^ = .00, number of letters *F*(1,18) = .39, *p* = .54, *η*^2^ = .021, number of phonological *F*(1,18) = 2.287, *p* = .14, *η*^2^ = .05 and orthographic neighbors *F*(1,18) = .11, *p* = .74, *η*^2^ = .006 and number of words that begin with the first two letters *F*(1,18) = .06, *p* = .81, *η*^2^ = .003, but differed in concreteness *F*(1,18) = 10.28, *p* = .005, *η*^2^ = .364.

Ten items were also selected to conform L2 (see S1 Table). Semantic relations among items in each list and between lists were kept to a minimum.

#### Procedure

Participants performed the task in groups (1 to 6 students). All instructions and stimuli were shown using an LCD projector. First, L1 words were presented for study. Then, participants were given the cue to either forget the instructions (“please forget this list because it was a probe, the experimental list will now be presented”) or recall (“you have just studied a list of words, another list for study will be presented right now”), followed by the L2. Each word appeared for 4000 ms followed by a blank screen (2000 ms) in a fixed random order. Finally, participants were first asked to recall L1 items.

#### Design

The experiment used a 2 (AoA: early-acquired, late-acquired) x 2 (cue: forget, remember) between-participants factorial design.

### Results

A 2 (AoA: early, late) x 2 (cue: forget, remember) ANOVA on L1 recall showed a main effect of cue *F*(1,72) = 15.58, *p* < .0001, $\eta_{p}^{2}$ = .18 and AoA *F*(1,72) = 34.78, *p* < .0001, $\;\eta_{p}^{2}$ = .33. See Table 2 and S2 Table. More importantly, the interaction reached statistical significance *F*(1,72) = 5.36, *p* < .02, $\eta_{p}^{2}$ = .07. Thus, while a reliable DF effect was present in the late-acquisition condition with the forget group recalling fewer L1 items than the remember group, *F*(1,36) = 29.90, *p* < .0001, *η*^2^ = .45, this effect was absent in the early-acquired condition, *F*(1,36)<1, *η*^2^ = .027.

**Table 2:** List 1 and List 2 recall (standard deviations in brackets) in Experiment 2.

	Remember	Forget	DF Effect(Remember—Forget)
L1			
Late	.39 (.15)	.15 (.12)	.24
Early	.53 (.21)	.47 (.18)	.06
L2			
Late	.29 (.19)	.54 (.17)	-25
Early	.43 (.21)	.51 (22)	-8

Although our hypothesis focused on L1, for completeness we also performed analyses on L2 recall (see Table 2). The corresponding 2 (AoA) x 2 (cue) ANOVA showed cue to be statistically significant *F*(1,72) = 13.297, *p* < .02, $\eta_{p}^{2}$ = .16, indicating better recall in the forget condition (M = 52, SD = .19) than in the remember condition (M = .36, SD = .21). This effect was, however, modulated by the AoA x cue interaction *F*(1,72) = 3.803, *p* = .05, $\eta_{p}^{2}$ = .05, with better L2 recall in the forget group relative to the remember group in the late-acquired words condition, *F*(1,36) = 18.48, *p* < .0001, *η*^2^ = .339 but not in the early-acquired words condition, *F*(1,36) = 1.249, *p* < .271, *η*^2^ = .034. The main effect of AoA did not reach statistical significance *F*(1,72) = 1.576, *p* = .21, $\eta_{p}^{2}$ = .02. Thus, results from list 2 parallel those from List 1 indicating that forgetting of List 1 items in the late AoA condition produces better recall of List 2 by reducing proactive interference. Because early AoA list 1 words were not forgotten, the List 2 effect was not present for the early AoA condition.

### Discussion

Experiment 2 showed that early-acquired words are less forgettable than late-acquired ones. Instructions to forget elicited the standard directed-forgetting effect in the late-acquisition condition but had no effect in the early-acquisition condition.

Our findings thus suggest that AoA also plays a prominent role in directed forgetting. Previous research has shown that LM-DF depends upon encoding interfering new material after receiving the cue to forget [45]. Our finding of non-intentional forgetting for early-acquired words points to the fact that the very nature of List 1 can also determine the DF effect. Thus, early acquired words seem to be more difficult to forget than later acquired words. This pattern was evident when comparing List 1 recall in the forget and remember condition, but also when looking at List 2 recall where L2 was better recalled if proactive interference was reduced by forgetting List 1. Again, this was the case only for late acquired words.

## General Discussion

Both experiments have demonstrated the protective effect of age of acquisition against both incidental (Experiment 1) and intentional (Experiment 2) episodic forgetting. Whereas regular RIF and DF appeared when materials involving late-acquired words were presented, no reliable forgetting was observed with early-acquired words.

The observed advantage for early-acquired words in these two experiments are consistent with previous findings in other fields showing that AoA plays a relevant role in word reading, lexical decision tasks, picture naming, and face processing [4–6]. From this perspective, the greater resistance of early-acquired representations (relative to later acquired representations) to be forgotten could be explained by current models of AoA. For example, early-acquired words are thought to be more interconnected in semantic memory than late-acquired ones [9,10], which are thought to be more difficult to integrate once the first representations have been stored in the memory system [11] (see also [12–14] for a review and for alternative hypotheses). Knowledge integration, a process whereby related memories become interconnected in the brain, has a long history in psychology for its protective role from interference [32, 46–51]. In addition, recent studies on episodic memory consolidation has shown that memories which can be integrated into preexisting knowledge are more easily acquired and retrieved than difficult to integrate information [52–53]. In fact, congruent representations (representations that can be easily linked to previous knowledge) have been shown to become quickly independent of the hippocampus and rapidly consolidate in the neocortex [53]. From this perspective, if early-acquired words, which are said to be highly consolidated and more strongly interconnected in our memory system, are presented in a memory task, their episodic representations could quickly be integrated into preexisting knowledge and, consequently, would become less vulnerable to episodic forgetting than late-acquired words.

From this viewpoint, early-acquired words would be easier to integrate because their very highly interconnected representations in memory provide them with multiple paths to connect them to the presented episodic information. Interestingly, this highly interconnected nature of early acquired words, while conferring them an advantage in preventing forgetting, it might turn in disadvantage when the situation calls for distinctive processing. Contrary to integration, distinctiveness derives from the processing of differences relative to some context or background [54]. By the very nature of being more interconnected and easy to integrate, early acquired words will be less distinctive than late-acquired words and, in consequence, they might be less recallable in some memory tasks. In fact, this balance between integration and distinctiveness may explain results from previous memory studies which failed to show AoA effects [15,17] or observed an advantage for late-acquired words [16,18].

Whereas we posit here that AoA may modulate vulnerability to forgetting (incidental and intentional), a potential criticism of this interpretation is that our findings could be accounted for by concreteness. Because early-acquired words tend to be more concrete than late-acquired ones (see material sections), it could be concreteness, rather than AoA, the variable modulating forgetting in our experiments. To deal with this possibility we carried out additional item analyses to disentangle the concreteness effect from AoA (see S1 Table). Importantly, the analysis showed that RIF was still modulated by AoA (*p* = .005), which supports the idea that AoA plays a role in making words more or less prone to episodic forgetting.

In sum then, the results of our two experiments indicate that the special characteristics of the information acquired early in life prevent the episodic forgetting that emerges during competition. Interestingly this greater resistance to forgetting does generalize across tasks involving incidental and intentional forgetting.

## Supporting Information

S1 Table Lists of words presented in Experiments 1 and 2. (DOCX)

S2 Table Experiment 1 and 2 data. (XLSX)
