# Supplementary material for: Forgetting “Novel” but Not “Dragon”: The Role of Age of Acquisition on Intentional and Incidental Forgetting
Source: PLoS One. 2016 May 10;11(5):e0155110. doi: 10.1371/journal.pone.0155110 (PMC4862635; doi:10.1371/journal.pone.0155110)
Supplement: S1 Table — (DOCX) [file pone.0155110.s001.docx]

**Supporting Information**

S1 Table. Lists of words presented in Experiments 1 and 2.

| **Experiment** | **Type** | **Word** | **Age of Acquisition** | **Frequency** | **Letters** | **Phonological neighbors** | **Orthographic neighbors** | **Bigrams** | **Concreteness** |
| --- | --- | --- | --- | --- | --- | --- | --- | --- | --- |
| **Experiment 1** | **Late** | relato | 4.12 | 38.64 | 6 | 22 | 13 | 16,576 | 3.57 |
|  |  | república | 5.79 | 36.85 | 9 | 2 | 0 | 16,576 | 4.87 |
|  |  | reflexión | 5.06 | 44.41 | 9 | 6 | 0 | 16,576 | 2.92 |
|  |  | receta | 3.85 | 13.94 | 6 | 11 | 8 | 16,576 | 5.36 |
|  |  | reverendo | 5.44 | 4.78 | 9 | 3 | 2 | 16,576 | 5.20 |
|  |  | refrán | 4.48 | 6.77 | 6 | 2 | 1 | 16,576 | 5.62 |
|  |  | década | 4.29 | 45.01 | 6 | 8 | 0 | 128 | 5.40 |
|  |  | desastre | 4.32 | 21.31 | 8 | 1 | 0 | 17,859 | 4.43 |
|  |  | derecho | 3.84 | 144.99 | 7 | 6 | 2 | 17,859 | 3.71 |
|  |  | delicia | 4.62 | 4.58 | 7 | 2 | 1 | 17,859 | 3.91 |
|  |  | devoción | 5.54 | 14.14 | 8 | 0 | 0 | 17,859 | 4.38 |
|  |  | detener | 4.12 | 21.51 | 7 | 2 | 2 | 17,859 | 4.04 |
|  |  | cadáver | 4.64 | 42.82 | 7 | 0 | 0 | 6,815 | 5.51 |
|  |  | cautela | 5.6 | 8.56 | 7 | 5 | 2 | 6,815 | 4.17 |
|  |  | campaña | 5.08 | 75.68 | 7 | 11 | 7 | 6,815 | 4.09 |
|  |  | canon | 6.17 | 2.59 | 5 | 21 | 7 | 6,815 | 3.31 |
|  |  | capote | 4.72 | 3.78 | 6 | 13 | 7 | 6,815 | 5.53 |
|  |  | carmín | 5.08 | 2.79 | 6 | 5 | 1 | 6,815 | 5.43 |
|  |  | fábula | 4.08 | 6.17 | 6 | 10 | 2 | 29 | 4.95 |
|  |  | fantasía | 3.68 | 28.68 | 8 | 2 | 2 | 1,537 | 2.97 |
|  |  | favor | 4.16 | 37.04 | 5 | 8 | 2 | 1,537 | 4.32 |
|  |  | factura | 5.12 | 10.56 | 7 | 7 | 3 | 1,537 | 5.77 |
|  |  | farsa | 5.38 | 4.38 | 5 | 11 | 7 | 1,537 | 4.13 |
|  |  | fauna | 4.08 | 7.57 | 5 | 8 | 5 | 1,537 | 4.64 |
|  | **Early** | colocar | 3.36 | 21.91 | 7 | 9 | 4 | 14,501 | 3.93 |
|  |  | conductor | 3.49 | 24.3 | 9 | 3 | 1 | 14,501 | 4.73 |
|  |  | codo | 2.16 | 8.36 | 4 | 45 | 27 | 14,501 | 5.50 |
|  |  | cohete | 3 | 7.17 | 6 | 16 | 2 | 14,501 | 5.95 |
|  |  | cordón | 2.35 | 8.17 | 6 | 4 | 5 | 14,501 | 5.34 |
|  |  | coser | 3 | 6.37 | 5 | 13 | 9 | 14,501 | 5.59 |
|  |  | mejilla | 3.4 | 18.52 | 7 | 7 | 4 | 2,819 | 6.57 |
|  |  | melena | 2.58 | 13.74 | 6 | 10 | 8 | 2,819 | 5.85 |
|  |  | meter | 2.16 | 25.89 | 5 | 15 | 8 | 2,819 | 4.35 |
|  |  | mechero | 3.29 | 4.38 | 7 | 7 | 4 | 2,819 | 6.65 |
|  |  | medalla | 3.12 | 8.17 | 7 | 2 | 1 | 2,819 | 5.63 |
|  |  | mermelada | 3 | 2.99 | 9 | 1 | 0 | 2,819 | 5.90 |
|  |  | camino | 2.32 | 229.44 | 6 | 10 | 9 | 6,815 | 4.75 |
|  |  | caballero | 3.17 | 37.84 | 9 | 9 | 3 | 6,815 | 5.42 |
|  |  | cariño | 2.81 | 28.08 | 6 | 6 | 3 | 6,815 | 3.72 |
|  |  | calendario | 2.91 | 15.73 | 10 | 1 | 0 | 6,815 | 6.18 |
|  |  | cachorro | 3.04 | 3.58 | 8 | 5 | 3 | 6,815 | 5.19 |
|  |  | cascabel | 2.96 | 1.99 | 8 | 0 | 0 | 6,815 | 5.84 |
|  |  | maestro | 2.14 | 46.6 | 7 | 5 | 3 | 5,502 | 5.59 |
|  |  | mapa | 2.68 | 24.9 | 4 | 42 | 24 | 5,502 | 4.83 |
|  |  | mañana | 2.44 | 284.61 | 6 | 5 | 3 | 5,502 | 4.86 |
|  |  | mago | 2.36 | 7.57 | 4 | 57 | 26 | 5,502 | 5.42 |
|  |  | mantel | 2.88 | 6.57 | 6 | 10 | 7 | 5,502 | 6.38 |
|  |  | margarita | 2.2 | 16.53 | 9 | 2 | 1 | 5,502 | 5.82 |
| **Experiment 2** | **Early List 1** | conductor | 3.49 | 24.3 | 9 | 3 | 1 | 14,501 | 4.73 |
|  |  | coser | 3 | 6.37 | 5 | 13 | 9 | 14,501 | 5.59 |
|  |  | cohete | 3 | 7.17 | 6 | 16 | 2 | 14,501 | 5.95 |
|  |  | mejilla | 3.4 | 18.52 | 7 | 7 | 4 | 2,819 | 6.57 |
|  |  | medalla | 3.12 | 8.17 | 7 | 2 | 1 | 2,819 | 5.63 |
|  |  | caballero | 3.17 | 37.84 | 9 | 9 | 3 | 6,815 | 5.42 |
|  |  | cachorro | 3.04 | 3.58 | 8 | 5 | 3 | 6,815 | 5.19 |
|  |  | mañana | 2.44 | 284.61 | 6 | 5 | 3 | 5,502 | 4.86 |
|  |  | margarita | 2.2 | 16.53 | 9 | 2 | 1 | 5,502 | 5.82 |
|  |  | mantel | 2.88 | 6.57 | 6 | 10 | 7 | 5,502 | 6.38 |
|  | **Late List 1** | refrán | 4.48 | 6.77 | 6 | 2 | 1 | 16,576 | 5.62 |
|  |  | república | 5.79 | 36.85 | 9 | 2 | 0 | 16,576 | 4.87 |
|  |  | reflexión | 5.06 | 44.41 | 9 | 6 | 0 | 16,576 | 2.92 |
|  |  | década | 4.29 | 45.01 | 6 | 8 | 0 | 128 | 5.40 |
|  |  | derecho | 3.84 | 144.99 | 7 | 6 | 2 | 17,859 | 3.71 |
|  |  | cautela | 5.6 | 8.56 | 7 | 5 | 2 | 6,815 | 4.17 |
|  |  | campaña | 5.08 | 75.68 | 7 | 11 | 7 | 6,815 | 4.09 |
|  |  | favor | 4.16 | 37.04 | 5 | 8 | 2 | 1,537 | 4.32 |
|  |  | farsa | 5.38 | 4.38 | 5 | 11 | 7 | 1,537 | 4.13 |
|  |  | factura | 5.12 | 10.56 | 7 | 7 | 3 | 1,537 | 5.77 |
|  | **List 2** | ajedrez | 3.29 | 15.8 | 7 | 1 | 1 | 258 | 5.80 |
|  |  | biografía | 5.08 | 14.4 | 9 | 3 | 0 | 742 | 4.76 |
|  |  | enigma | 5.29 | 11.4 | 6 | 1 | 0 | 9,672 | 3.48 |
|  |  | laberinto | 3.79 | 17.4 | 9 | 1 | 0 | 1,914 | 5.79 |
|  |  | negativo |  | 20.6 | 8 | 2 | 1 | 903 |  |
|  |  | péndulo |  | 7.6 | 7 | 2 | 1 | 79 | 5.38 |
|  |  | peregrino | 5.16 | 10 | 9 | 5 | 4 | 4,343 | 5.90 |
|  |  | salvaje |  | 19.6 | 7 | 9 | 2 | 3,056 | 4.21 |
|  |  | veneno | 3.72 | 12.6 | 6 | 5 | 2 | 1,827 | 4.48 |
|  |  | zapato | 1.72 | 14.6 | 6 | 5 | 1 | 677 | 6.48 |

Age of acquisition, frequency, number of letters, number of phonological and orthographic neighbors, number of words that begin with the same first bigram, and concreteness scores for Experiment 1 and Experiment 2 items. Given that there were differences in concreteness between early- and late-acquired lists, we performed item analyses on the data from Experiment 1 (where more items were included) by introducing concreteness as a covariate and AoA and item type as independent variables.. Results showed that, once eliminated the effect of concreteness, RIF was still modulated by AoA *F*(1,21) = 9.792, *p = .*005*,* $\eta_{p}^{2}$= .32. Neither concreteness *F*(1,21) = 0.9, *p = .*35*,* $\eta_{p}^{2}$= .04, nor the interaction concreteness x type of item *F*(1,21) = 1.44, *p =* .24*,* $\eta_{p}^{2}$= .06 had an effect on RIF.
